# The genome sequence of the Rock Grayling, *Hipparchia semele *(Linnaeus, 1758)

**DOI:** 10.12688/wellcomeopenres.20183.1

**Published:** 2023-10-26

**Authors:** Callum J. Macgregor, Ilik J. Saccheri, Wellcome Sanger Institute Tree of Life programme, Wellcome Sanger Institute Scientific Operations: DNA Pipelines collective, Tree of Life Core Informatics collective, Darwin Tree of Life Consortium

**Affiliations:** 1 Leverhulme Centre for Anthropocene Biodiversity, University of York, York, England, UK; 2 British Trust for Ornithology, Bangor, Wales, UK; 3 Department of Evolution, Ecology and Behaviour, University of Liverpool, Liverpool, England, UK

**Keywords:** Hipparchia semele, Rock Grayling, genome sequence, chromosomal, Lepidoptera

## Abstract

We present a genome assembly from an individual female *Hipparchia semele* (the Rock Grayling; Arthropoda; Insecta; Lepidoptera; Nymphalidae). The genome sequence is 403.4 megabases in span. Most of the assembly is scaffolded into 30 chromosomal pseudomolecules, including the W and Z sex chromosomes. The mitochondrial genome has also been assembled and is 15.22 kilobases in length. Gene annotation of this assembly on Ensembl identified 17,540 protein coding genes.

## Species taxonomy

Eukaryota; Metazoa; Eumetazoa; Bilateria; Protostomia; Ecdysozoa; Panarthropoda; Arthropoda; Mandibulata; Pancrustacea; Hexapoda; Insecta; Dicondylia; Pterygota; Neoptera; Endopterygota; Amphiesmenoptera; Lepidoptera; Glossata; Neolepidoptera; Heteroneura; Ditrysia; Obtectomera; Papilionoidea; Nymphalidae; Satyrinae; Satyrini; Satyrina; *Hipparchia*; *Hipparchia semele* (Linnaeus, 1758) (NCBI:txid111912).

## Background

**Figure 1.**
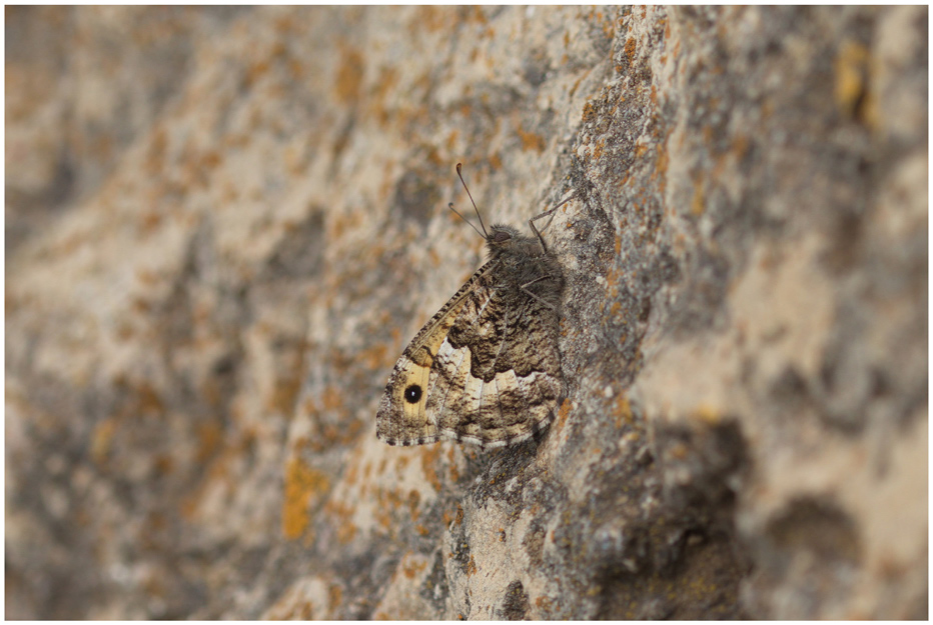
Image of *Hipparchia semele* (not the specimen used for genome sequencing). Photograph by Callum Macgregor.

The Grayling (or Rock Grayling), *Hipparchia semele* (Linnaeus, 1758) ( Figure 1), is a medium-sized xerothermophilous butterfly in the family Nymphalidae, occupying habitats with dry or rapidly draining soils, especially heathlands and coastal sand dunes, but also post-industrial sites ( Tropek *et al.*, 2017). It is broadly distributed across much of Europe, but has a primarily coastal distribution in northerly countries including the UK. It is declining across most of its range ( Tropek *et al.*, 2017), with habitat loss and fragmentation considered to be among the important anthropogenic drivers of change ( De Ro *et al.*, 2021). It has a fragmented distribution within its range and exhibits metapopulation dynamics ( van Strien *et al.*, 2011). Colonies can vary in size from tens of individuals to several thousand; the Darwin Tree of Life Project genome assembly presented here is from a specimen sourced from one of the largest UK colonies, on heathland in the New Forest, Hampshire.

Multiple races or subspecies of *H. semele* have been described within Britain alone ( Eeles, 2019). Most notably, the population on the Great Orme, North Wales is distinct in both appearance and phenology ( Dapporto *et al.*, 2019; Middlebrook *et al.*, 2019; Thompson, 1944). Variation in the size and position of wing ocelli (spots) is thought to be adaptive, and related to predator defence ( Dapporto *et al.*, 2019). Evidence also exists for local adaptation to climate ( Roy *et al.*, 2015), but the species has not advanced its phenology in recent years ( Macgregor *et al.*, 2019). The chromosome number described for a Finnish population is 29 ( Federley, 1938), which is consistent with the number in the present genome assembly.

## Genome sequence report

The genome was sequenced from one female *Hipparchia semele* collected from Beaulieu Heath, England (50.80, –1.50). A total of 58-fold coverage in Pacific Biosciences single-molecule HiFi long reads was generated. Primary assembly contigs were scaffolded with chromosome conformation Hi-C data. Manual assembly curation corrected 16 missing joins or mis-joins and removed one haplotypic duplication, reducing the assembly length by 1.23% and the scaffold number by 7.69% and decreasing the scaffold N50 by 1.04%.

**Figure 2.**
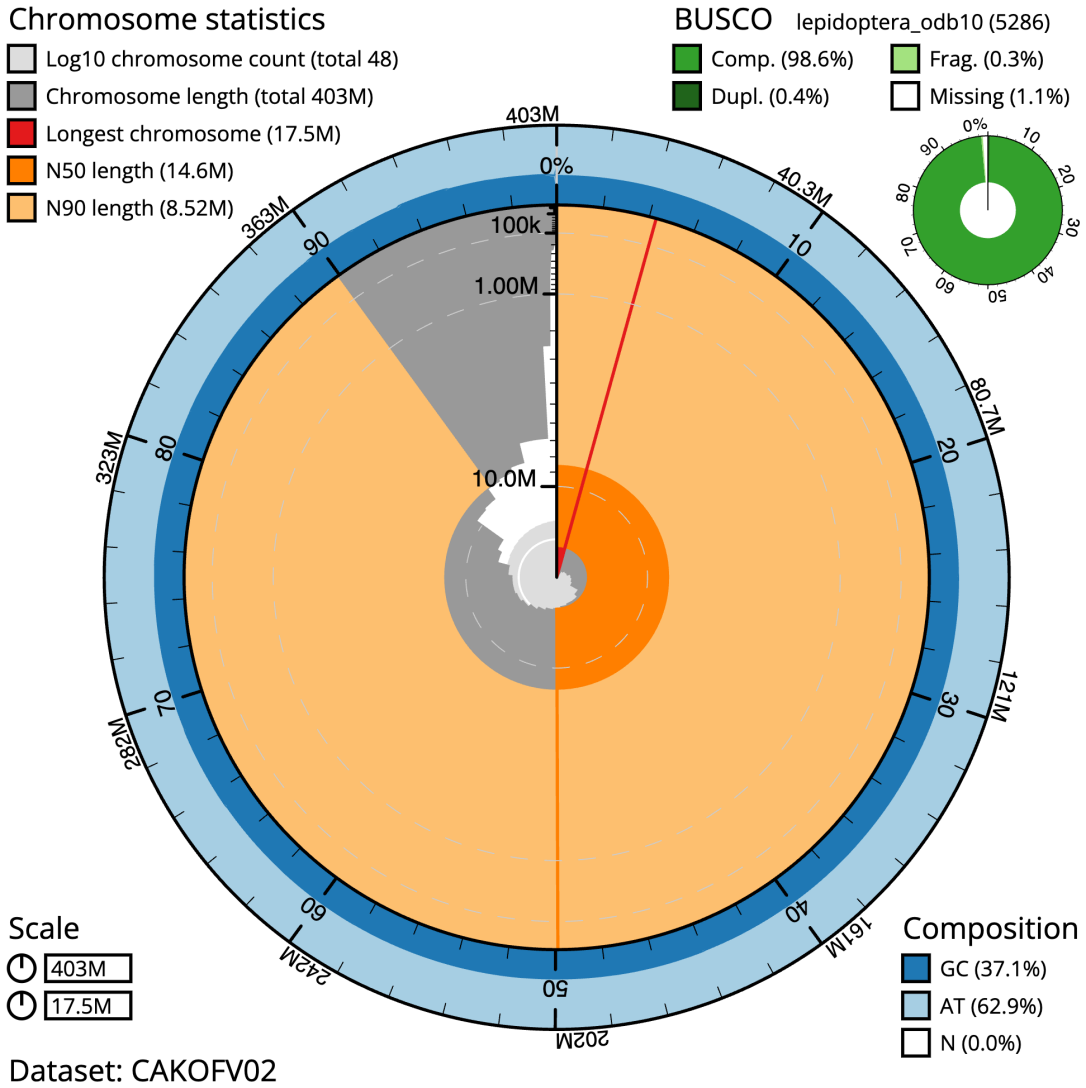
Genome assembly of *Hipparchia semele*, ilHipSeme1.2: metrics. The BlobToolKit Snailplot shows N50 metrics and BUSCO gene completeness. The main plot is divided into 1,000 size-ordered bins around the circumference with each bin representing 0.1% of the 403,420,717 bp assembly. The distribution of scaffold lengths is shown in dark grey with the plot radius scaled to the longest scaffold present in the assembly (17,487,639 bp, shown in red). Orange and pale-orange arcs show the N50 and N90 scaffold lengths (14,619,000 and 8,523,649 bp), respectively. The pale grey spiral shows the cumulative scaffold count on a log scale with white scale lines showing successive orders of magnitude. The blue and pale-blue area around the outside of the plot shows the distribution of GC, AT and N percentages in the same bins as the inner plot. A summary of complete, fragmented, duplicated and missing BUSCO genes in the lepidoptera_odb10 set is shown in the top right. An interactive version of this figure is available at https://blobtoolkit.genomehubs.org/view/ilHipSeme1.2/dataset/CAKOFV02/snail.

The final assembly has a total length of 403.4 Mb in 47 sequence scaffolds with a scaffold N50 of 14.6 Mb ( Table 1). Most (99.89%) of the assembly sequence was assigned to 30 chromosomal-level scaffolds, representing 28 autosomes and the W and Z sex chromosomes. Chromosome-scale scaffolds confirmed by the Hi-C data are named in order of size ( Figure 2– Figure 5; Table 2). While not fully phased, the assembly deposited is of one haplotype. Contigs corresponding to the second haplotype have also been deposited. The mitochondrial genome was also assembled and can be found as a contig within the multifasta file of the genome submission.

**Table 1.** Genome data for *Hipparchia semele*, ilHipSeme1.2.

Project accession data		
Assembly identifier	ilHipSeme1.2	
Assembly release data	2022-03-23	
Species	*Hipparchia semele*	
Specimen	ilHipSeme1	
NCBI taxonomy ID	111912	
BioProject	PRJEB50742	
BioSample ID	SAMEA9252595	
Isolate information	ilHipSeme1, female: whole organism (DNA sequencing) ilHipSeme2, female: abdomen (Hi-C scaffolding)	
Assembly metrics *		*Benchmark*
Consensus quality (QV)	68	*≥ 50*
*k*-mer completeness	100%	*≥ 95%*
BUSCO **	C:98.6%[S:98.1%,D:0.4%],F:0.3%, M:1.1%,n:5,286	*C ≥ 95%*
Percentage of assembly mapped to chromosomes	99.89%	*≥ 95%*
Sex chromosomes	W and Z chromosomes	*localised homologous pairs*
Organelles	Mitochondrial genome assembled	*complete single alleles*
Raw data accessions		
PacificBiosciences SEQUEL II	ERR8575378	
Hi-C Illumina	ERR8571664	
Genome assembly		
Assembly accession	GCA_933228805.2	
*Accession of alternate haplotype*	GCA_933228805.1	
Span (Mb)	403.4	
Number of contigs	61	
Contig N50 length (Mb)	14.5	
Number of scaffolds	47	
Scaffold N50 length (Mb)	14.6	
Longest scaffold (Mb)	17.5	
Genome annotation		
Number of protein-coding genes	17,540	
Number of gene transcripts	17,738	

* Assembly metric benchmarks are adapted from column VGP-2020 of “Table 1: Proposed standards and metrics for defining genome assembly quality” from ( Rhie *et al.*, 2021). ** BUSCO scores based on the lepidoptera_odb10 BUSCO set using v5.3.2. C = complete [S = single copy, D = duplicated], F = fragmented, M = missing, n = number of orthologues in comparison. A full set of BUSCO scores is available at https://blobtoolkit.genomehubs.org/view/ilHipSeme1.2/dataset/CAKOFV02/busco.

**Figure 3.**
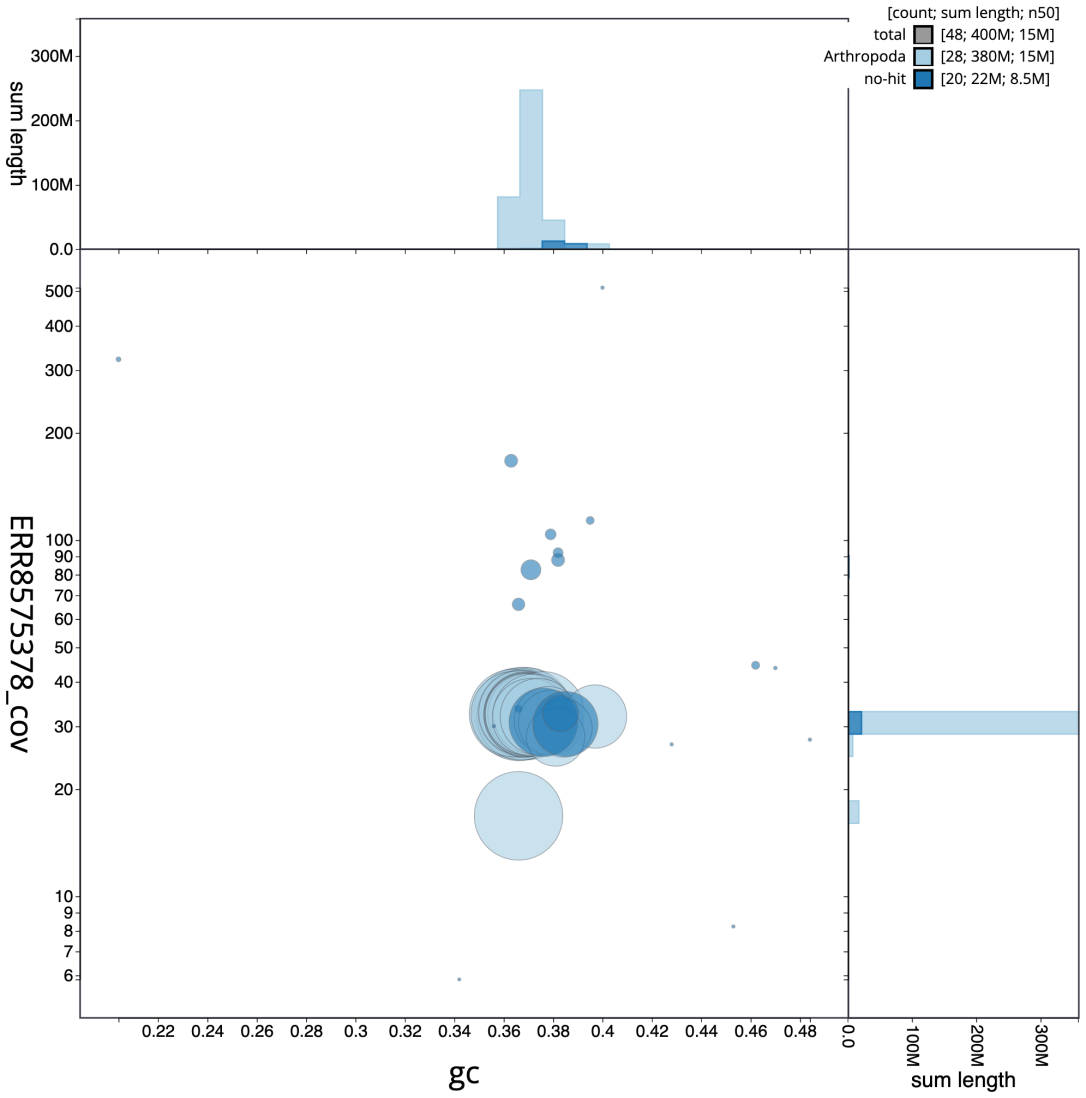
Genome assembly of *Hipparchia semele*, ilHipSeme1.2: BlobToolKit GC-coverage plot. Scaffolds are coloured by phylum. Circles are sized in proportion to scaffold length. Histograms show the distribution of scaffold length sum along each axis. An interactive version of this figure is available at https://blobtoolkit.genomehubs.org/view/ilHipSeme1.2/dataset/CAKOFV02/blob.

**Figure 4.**
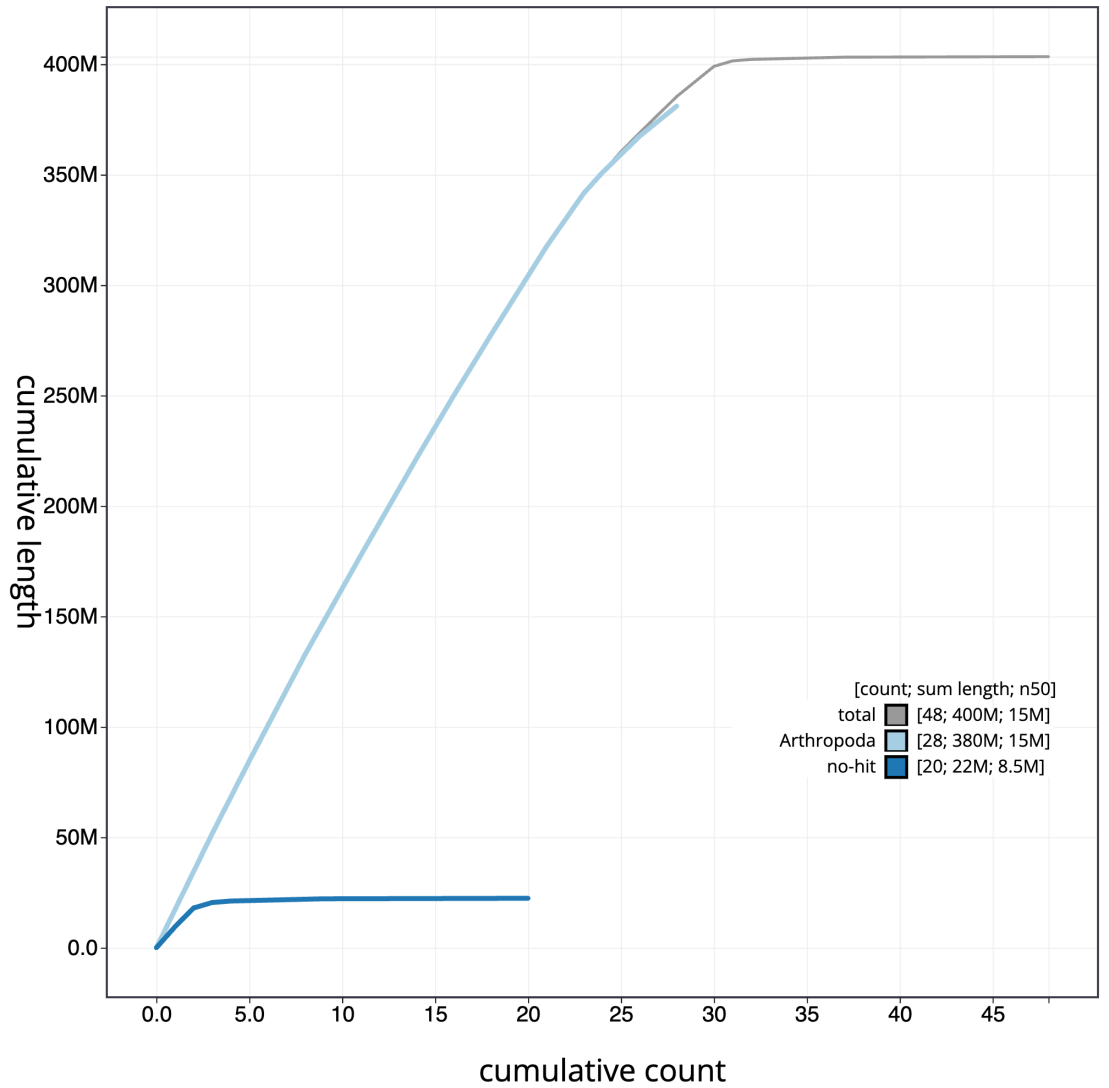
Genome assembly of *Hipparchia semele*, ilHipSeme1.2: BlobToolKit cumulative sequence plot. The grey line shows cumulative length for all scaffolds. Coloured lines show cumulative lengths of scaffolds assigned to each phylum using the buscogenes taxrule. An interactive version of this figure is available at https://blobtoolkit.genomehubs.org/view/ilHipSeme1.2/dataset/CAKOFV02/cumulative.

**Figure 5.**
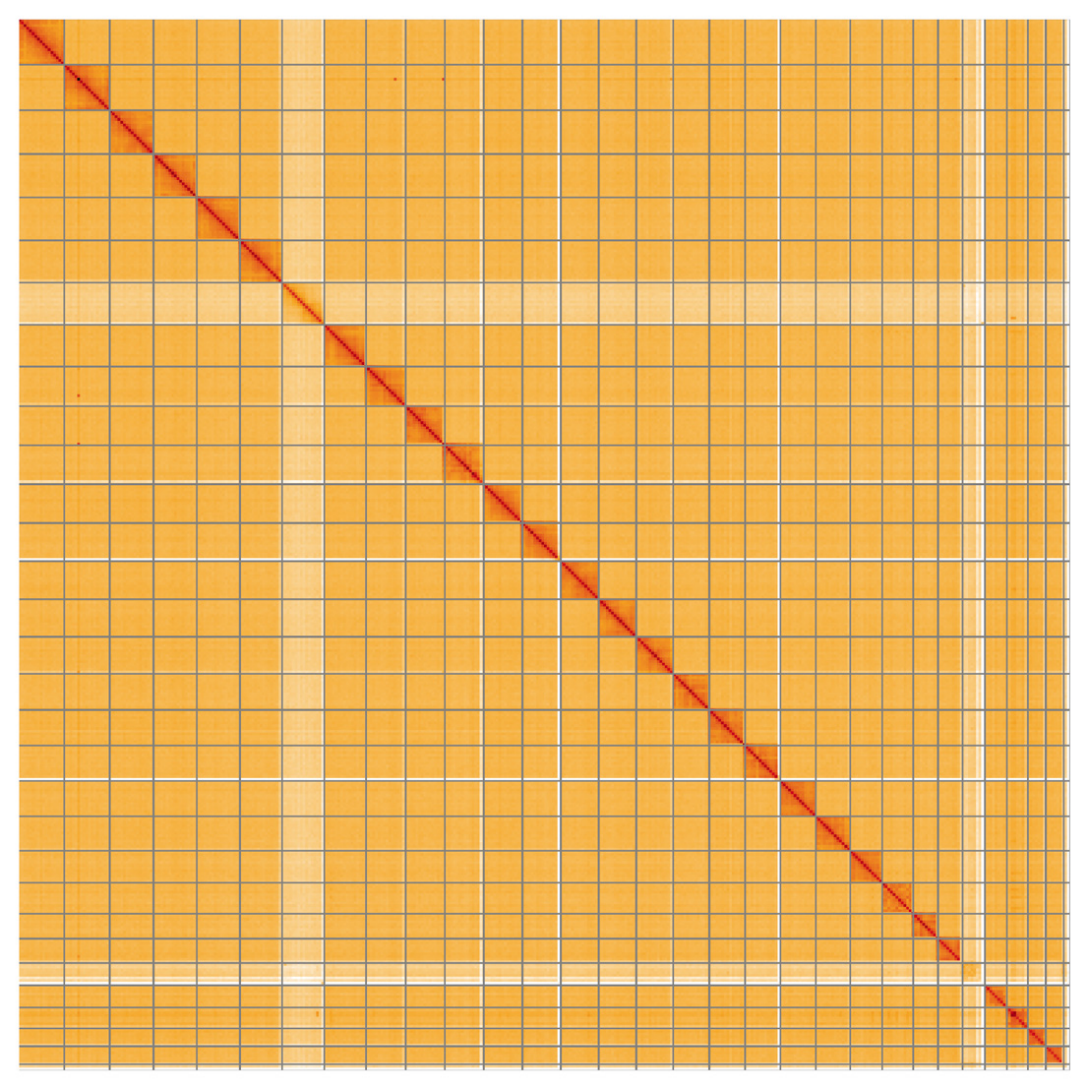
Genome assembly of *Hipparchia semele*, ilHipSeme1.2: Hi-C contact map of the ilHipSeme1.2 assembly, visualised using HiGlass. Chromosomes are shown in order of size from left to right and top to bottom. An interactive version of this figure may be viewed at https://genome-note-higlass.tol.sanger.ac.uk/l/?d=dbfZsqrJQlqmsZnT7Knz0w.

**Table 2.** Chromosomal pseudomolecules in the genome assembly of *Hipparchia semele*, ilHipSeme1.

INSDC accession	Chromosome	Length (Mb)	GC%
OW121709.1	1	17.49	37.0
OW121710.1	2	17.41	36.5
OW121711.1	3	16.71	36.5
OW121712.1	4	16.53	37.0
OW121713.1	5	16.44	36.5
OW121714.1	6	16.13	36.5
OW121715.1	7	15.95	36.5
OW121716.1	8	15.18	36.5
OW121717.1	9	14.88	37.0
OW121718.1	10	14.87	36.5
OW121719.1	11	14.77	37.0
OW121720.1	12	14.62	36.5
OW121721.1	13	14.52	37.0
OW121722.1	14	14.33	37.0
OW121723.1	15	14.09	37.0
OW121724.1	16	13.82	37.0
OW121725.1	17	13.62	37.5
OW121726.1	18	13.5	37.0
OW121727.1	19	13.49	37.0
OW121728.1	20	13.16	37.0
OW121729.1	21	12.2	37.0
OW121730.1	22	11.84	37.5
OW121731.1	23	9.46	37.5
OW121732.1	24	9.34	38.0
OW121733.1	25	8.39	38.0
OW121734.1	26	8.05	39.5
OW121736.1	28	6.82	38.0
OW121735.1	27	6.82	38.5
OW121738.1	W	8.52	38.5
OW121737.1	Z	16.05	36.5
OW121739.2	MT	0.02	20.5

The estimated Quality Value (QV) of the final assembly is 68 with *k*-mer completeness of 100%, and the assembly has a BUSCO v5.3.2 completeness of 98.6% (single = 98.1%, duplicated = 0.4%), using the lepidoptera_odb10 reference set ( *n* = 5,286).

Metadata for specimens, spectral estimates, sequencing runs, contaminants and pre-curation assembly statistics can be found at https://links.tol.sanger.ac.uk/species/111912.

## Genome annotation report

The *Hipparchia semele* genome assembly (GCA_933228805.2) was annotated using the Ensembl rapid annotation pipeline ( Table 1; https://rapid.ensembl.org/Hipparchia_semele_GCA_933228805.1/Info/Index). The resulting annotation includes 17,738 transcribed mRNAs from 17,540 protein-coding genes.

## Methods

### Sample acquisition and nucleic acid extraction

Two female *Hipparchia semele* specimens were collected with permission from Beaulieu Heath, England, UK (latitude 50.80, longitude –1.50) on 2017-07-06 using a sweep net. The specimens were collected by Callum Macgregor (University of York) and formally identified by Ilik Saccheri (University of Liverpool) and stored at –80°C. One specimen (specimen ID SAN0001387, individual ilHipSeme1) was used for DNA sequencing, and a second specimen (specimen ID SAN0001388, individual ilHipSeme2) was used for Hi-C scaffolding.

DNA was extracted at the Tree of Life laboratory, Wellcome Sanger Institute (WSI). The ilHipSeme1 sample was weighed and dissected on dry ice with tissue set aside for Hi-C sequencing. Tissue from the whole organism was cryogenically disrupted to a fine powder using a Covaris cryoPREP Automated Dry Pulveriser, receiving multiple impacts. High molecular weight (HMW) DNA was extracted using the Qiagen MagAttract HMW DNA extraction kit. HMW DNA was sheared into an average fragment size of 12–20 kb in a Megaruptor 3 system with speed setting 30. Sheared DNA was purified by solid-phase reversible immobilisation using AMPure PB beads with a 1.8X ratio of beads to sample to remove the shorter fragments and concentrate the DNA sample. The concentration of the sheared and purified DNA was assessed using a Nanodrop spectrophotometer and Qubit Fluorometer and Qubit dsDNA High Sensitivity Assay kit. Fragment size distribution was evaluated by running the sample on the FemtoPulse system.

### Sequencing

Pacific Biosciences HiFi circular consensus ud DNA sequencing libraries were constructed according to the manufacturers’ instructions. DNA sequencing was performed by the Scientific Operations core at the WSI on a Pacific Biosciences SEQUEL II (HiFi) instrument. Hi-C data were also generated from abdomen tissue of ilHipSeme2 using the Arima2 kit and sequenced on the Illumina NovaSeq 6000 instrument.

### Genome assembly, curation and evaluation

Assembly was carried out with Hifiasm ( Cheng *et al.*, 2021) and haplotypic duplication was identified and removed with purge_dups ( Guan *et al.*, 2020). The assembly was then scaffolded with Hi-C data ( Rao *et al.*, 2014) using YaHS ( Zhou *et al.*, 2023). The assembly was checked for contamination and corrected using the gEVAL system ( Chow *et al.*, 2016) as described previously ( Howe *et al.*, 2021). Manual curation was performed using gEVAL, HiGlass ( Kerpedjiev *et al.*, 2018) and Pretext ( Harry, 2022). The mitochondrial genome was assembled using MitoHiFi ( Uliano-Silva *et al.*, 2023), which runs MitoFinder ( Allio *et al.*, 2020) or MITOS ( Bernt *et al.*, 2013) and uses these annotations to select the final mitochondrial contig and to ensure the general quality of the sequence.

A Hi-C map for the final assembly was produced using bwa-mem2 ( Vasimuddin *et al.*, 2019) in the Cooler file format ( Abdennur & Mirny, 2020). To assess the assembly metrics, the *k*-mer completeness and QV consensus quality values were calculated in Merqury ( Rhie *et al.*, 2020). This work was done using Nextflow ( Di Tommaso *et al.*, 2017) DSL2 pipelines “sanger-tol/readmapping” ( Surana *et al.*, 2023a) and “sanger-tol/genomenote” ( Surana *et al.*, 2023b). The genome was analysed within the BlobToolKit environment ( Challis *et al.*, 2020) and BUSCO scores ( Manni *et al.*, 2021; Simão *et al.*, 2015) were calculated.

Table 3 contains a list of relevant software tool versions and sources.

**Table 3.** Software tools: versions and sources.

Software tool	Version	Source
BlobToolKit	4.0.7	https://github.com/blobtoolkit/blobtoolkit
BUSCO	5.3.2	https://gitlab.com/ezlab/busco
gEVAL	N/A	https://geval.org.uk/
Hifiasm	0.16.1-r375	https://github.com/chhylp123/hifiasm
HiGlass	1.11.6	https://github.com/higlass/higlass
Merqury	MerquryFK	https://github.com/thegenemyers/MERQURY.FK
MitoHiFi	2	https://github.com/marcelauliano/MitoHiFi
PretextView	0.2	https://github.com/wtsi-hpag/PretextView
purge_dups	1.2.3	https://github.com/dfguan/purge_dups
sanger-tol/genomenote	v1.0	https://github.com/sanger-tol/genomenote
sanger-tol/readmapping	1.1.0	https://github.com/sanger-tol/readmapping/tree/1.1.0
YaHS	yahs-1.1.91eebc2	https://github.com/c-zhou/yahs

### Genome annotation

The BRAKER2 pipeline ( Brůna *et al.*, 2021) was used in the default protein mode to generate annotation for the *Hipparchia semele* assembly (GCA_933228805.2) in Ensembl Rapid Release.

### Wellcome Sanger Institute – Legal and Governance

The materials that have contributed to this genome note have been supplied by a Darwin Tree of Life Partner. The submission of materials by a Darwin Tree of Life Partner is subject to the **‘Darwin Tree of Life Project Sampling Code of Practice’**, which can be found in full on the Darwin Tree of Life website here. By agreeing with and signing up to the Sampling Code of Practice, the Darwin Tree of Life Partner agrees they will meet the legal and ethical requirements and standards set out within this document in respect of all samples acquired for, and supplied to, the Darwin Tree of Life Project.

Further, the Wellcome Sanger Institute employs a process whereby due diligence is carried out proportionate to the nature of the materials themselves, and the circumstances under which they have been/are to be collected and provided for use. The purpose of this is to address and mitigate any potential legal and/or ethical implications of receipt and use of the materials as part of the research project, and to ensure that in doing so we align with best practice wherever possible. The overarching areas of consideration are:

•   Ethical review of provenance and sourcing of the material

•   Legality of collection, transfer and use (national and international)

Each transfer of samples is further undertaken according to a Research Collaboration Agreement or Material Transfer Agreement entered into by the Darwin Tree of Life Partner, Genome Research Limited (operating as the Wellcome Sanger Institute), and in some circumstances other Darwin Tree of Life collaborators.

## Data Availability

European Nucleotide Archive: *Hipparchia semele* (rock grayling). Accession number PRJEB50742; https://identifiers.org/ena.embl/PRJEB50742. ( Wellcome Sanger Institute, 2022) The genome sequence is released openly for reuse. The *Hipparchia semele* genome sequencing initiative is part of the Darwin Tree of Life (DToL) project. All raw sequence data and the assembly have been deposited in INSDC databases. Raw data and assembly accession identifiers are reported in Table 1.

## Author information

Members of the Wellcome Sanger Institute Tree of Life programme are listed here: https://doi.org/10.5281/zenodo.4783585.

Members of Wellcome Sanger Institute Scientific Operations: DNA Pipelines collective are listed here: https://doi.org/10.5281/zenodo.4790455.

Members of the Tree of Life Core Informatics collective are listed here: https://doi.org/10.5281/zenodo.5013541.

Members of the Darwin Tree of Life Consortium are listed here: https://doi.org/10.5281/zenodo.4783558.
