# Supplementary material for: The genome sequence of the Rock Grayling, Hipparchia semele (Linnaeus, 1758)
Source: Wellcome Open Res. 2023 Oct 26;8:495. [Version 1] doi: 10.12688/wellcomeopenres.20183.1 (PMC10825418; doi:10.12688/wellcomeopenres.20183.1)
Supplement: Peer review history

# Reviewer report: Reviewer response for version 1

**DOI:** 10.21956/wellcomeopenres.22346.r71644

**Published:** 2024-01-29

**Authors:** Daniel Berner

**Affiliations:** 1 University of Basel, Basel, Switzerland

Magregor and Saccheri here present a genome assembly for the Nymphalid butterfly *Hipparchia semele*. Based on PacBio HiFi long-read sequencing and Hi-C scaffolding, they achieve a top-quality assembly, which is then annotated using a standard pipeline. The methods are generally well described, and the results clearly presented. This new assembly certainly provides a valuable resource for future investigations.

 I just have three minor suggestions:

 1) ‘Variation in the size and position of wing ocelli (spots)’: one could be more precise by using ‘eye spots’ here; I guess this is what you refer to, right?

 2) ‘related to predator defence’: perhaps ‘predator avoidance’ is better?

 3) It is not fully clear to me which individual was used for Hi-C. First, you specify that ‘One specimen (specimen ID SAN0001387, individual ilHipSeme1) was used for DNA sequencing, and a second specimen (specimen ID SAN0001388, individual ilHipSeme2) was used for Hi-C scaffolding.’ But in the subsequent paragraph you state that ‘The ilHipSeme1 sample was weighed and dissected on dry ice with tissue set aside for Hi-C sequencing’. So the first individual from above was also used for Hi-C? If this is true, I would suggest writing ‘One specimen (specimen ID SAN0001387, individual ilHipSeme1) was used for DNA sequencing and Hi-C scaffolding, and a second specimen (specimen ID SAN0001388, individual ilHipSeme2) was used for Hi-C scaffolding only.’

Are sufficient details of methods and materials provided to allow replication by others?

Partly

Is the rationale for creating the dataset(s) clearly described?

Yes

Are the datasets clearly presented in a useable and accessible format?

Yes

Are the protocols appropriate and is the work technically sound?

Yes

Reviewer Expertise:

Evolutionary ecology; Population genomics; Adaptive diversification

I confirm that I have read this submission and believe that I have an appropriate level of expertise to confirm that it is of an acceptable scientific standard.

# Reviewer report: Reviewer response for version 1

**Published:** 2024-01-23

**Authors:** Huateng Huang, Delong Guan

**Affiliations:** 1 Shaanxi Normal University, Xi’an, China; 2 college of chemistry and biological engineering, Hechi University (Ringgold ID: 56655), Yizhou, Guangxi, China

Dear authors,

 I have reviewed your manuscript titled "The genome sequence of the Rock Grayling, Hipparchia semele (Linnaeus, 1758)" submitted to Wellcome Open Research with great interest. This data report presents the 403.4 Mb draft genome assembly of the grayling butterfly Hipparchia semele, which was generated using Pacific Biosciences long reads and scaffolded with Hi-C data. Seventeen thousand five hundred forty protein-coding genes were annotated. This concise and well-written manuscript describes valuable genomic resources for an ecologically critical lepidopteran species. I only have several minor comments and suggestions:

  

- Page 3, third line on the right: "collected from Beaulieu Heath, England (50.80, –1.50)." are the numbers latitude and longitude? Please provide the unit.
- Details on heterozygosity and phasing of the assembly are lacking. As this is from a wild individual, heterozygosity is expected to be substantial. Please provide parameters of critical steps, like purge_dups, to ensure reproducibility of the study. Also, the parameters for Hifisam and the Hi-C scaffolding process before using yahs.
- Tools like bwa/chromap used for reading mapping and interaction calling are not listed in Table 3.
- Page 5, under "Sequencing," "Pacific Biosciences HiFi circular consensus ud DNA"
- It is unclear to me what the y-axis label "ERRxxx_cov" means in Figure 3.

Sincerely,

 Huateng Huang and De-Long Guan

Are sufficient details of methods and materials provided to allow replication by others?

Yes

Is the rationale for creating the dataset(s) clearly described?

Yes

Are the datasets clearly presented in a useable and accessible format?

Yes

Are the protocols appropriate and is the work technically sound?

Yes

Reviewer Expertise:

phylogenomics

We confirm that we have read this submission and believe that we have an appropriate level of expertise to confirm that it is of an acceptable scientific standard.
